# Supplementary material for: Synergistic celecoxib and dimethyl-celecoxib combinations block cervix cancer growth through multiple mechanisms
Source: PLoS One. 2024 Sep 26;19(9):e0308233. doi: 10.1371/journal.pone.0308233 (PMC11426494; doi:10.1371/journal.pone.0308233)
Supplement: S1 Fig — Effect of CXB and DMC combinations on cell cycle phases in HeLa (A) and SiHa (B) cells. The indicated drugs were added at the following concentrations: CXB (5 μM); CXB (5 μM)/CP (2 μM); CXB (5 μM)/PA (15 μM); DMC (15 μM), DMC (15 μM)/CP (5 μM) or DMC (15 μM)/PA (20 μM). Cells were exposed to the drugs for 24 h. Data shown represent the mean ± S.D. of at least three different preparations. *p < 0.05 vs. control (non-treated cells). (DOCX) [file pone.0308233.s001.docx]

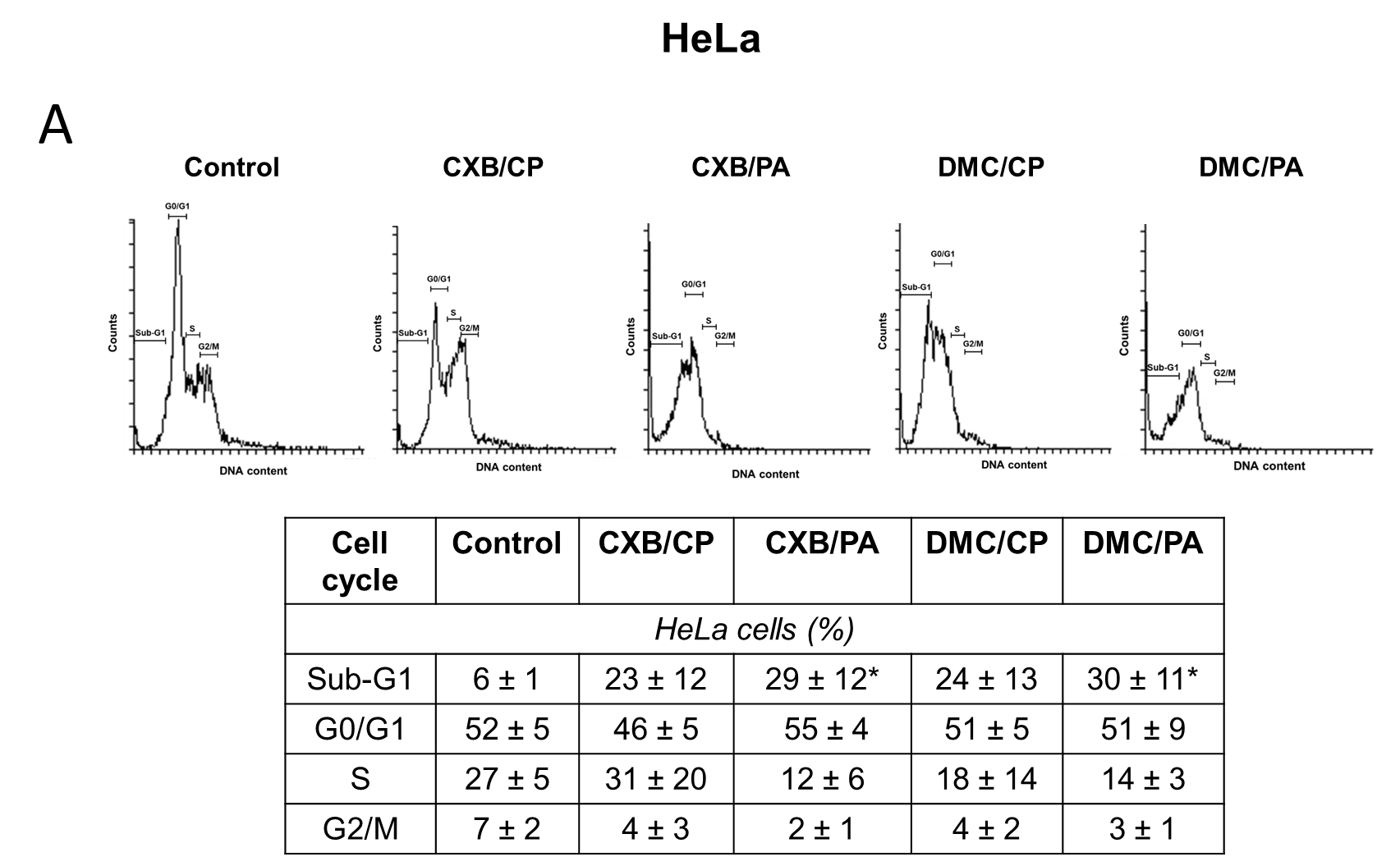


**
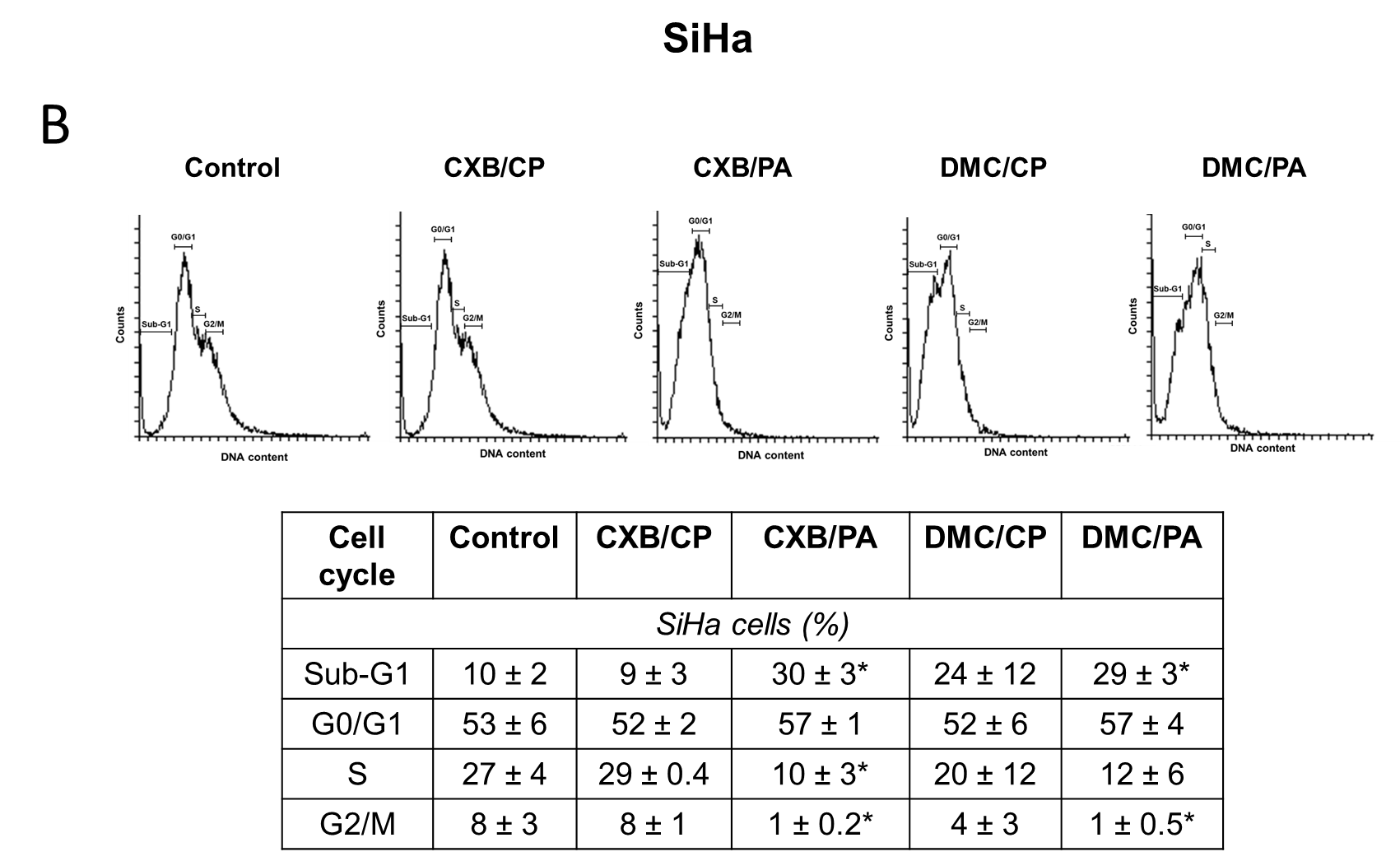
**

**S1 Fig. Effect of CXB and DMC combinations on cell cycle phases in HeLa (A) and SiHa (B) cells.** The indicated drugs were added at the following concentrations: CXB (5 µM); CXB (5 µM)/CP (2 µM); CXB (5 µM)/PA (15 µM); DMC (15 µM), DMC (15 µM)/CP (5 µM) or DMC (15 µM)/PA (20 µM). Cells were exposed to the drugs for 24 h. Data shown represent the mean ± S.D. of at least three different preparations. *p < 0.05 *vs*. control (non-treated cells).
