# Supplementary material for: Synergistic celecoxib and dimethyl-celecoxib combinations block cervix cancer growth through multiple mechanisms
Source: PLoS One. 2024 Sep 26;19(9):e0308233. doi: 10.1371/journal.pone.0308233 (PMC11426494; doi:10.1371/journal.pone.0308233)
Supplement: S2 Fig — Data shown represent the mean ± S.D. of at least three different preparations. *p < 0.05 vs. control (non-treated cells); **p < 0.05 vs. CXB. (DOCX) [file pone.0308233.s002.docx]

**
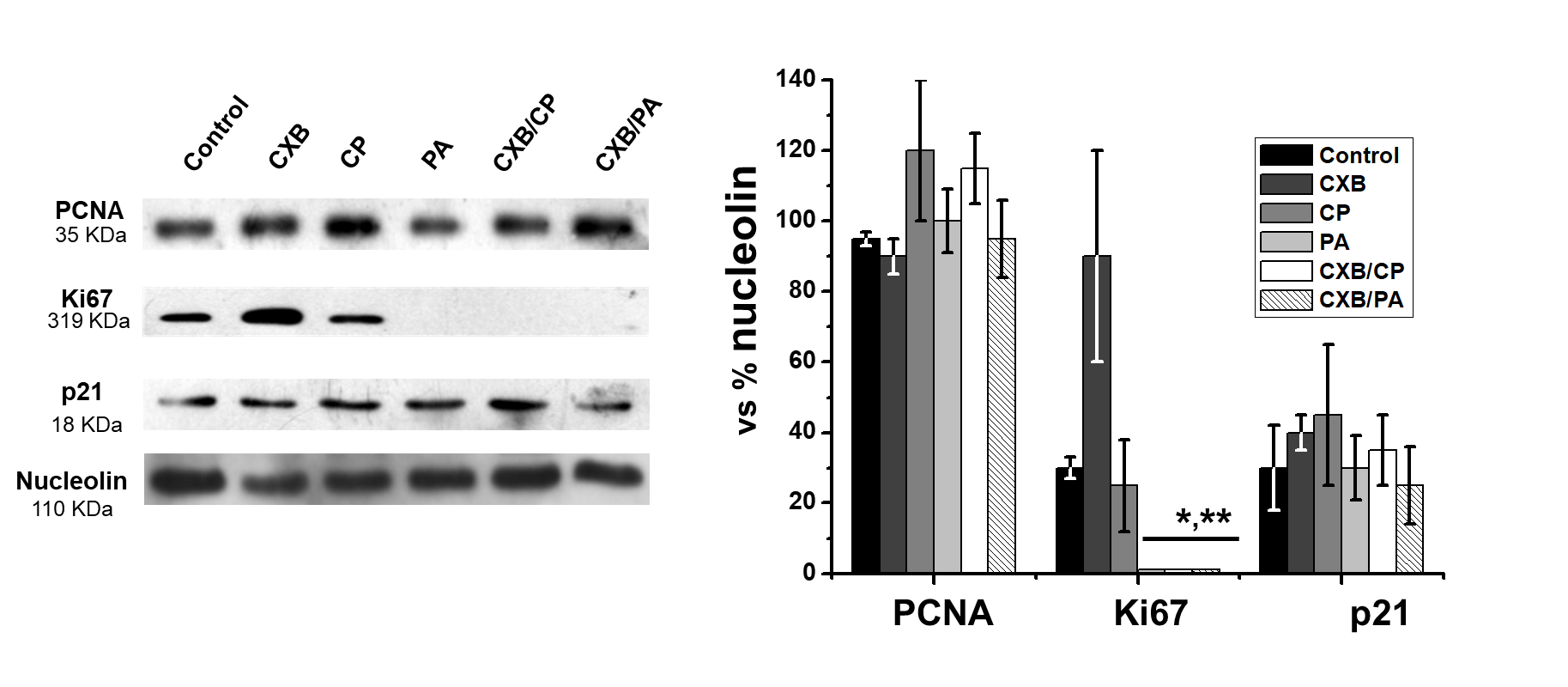
**

**S2 Fig.** **Proliferation marker protein contents in CXB treated-HeLa cells**. Data shown represent the mean ± S.D. of at least three different preparations. ^*^p < 0.05 *vs*. control (non-treated cells); **p < 0.05 *vs*. CXB.
