# Supplementary material for: Synergistic celecoxib and dimethyl-celecoxib combinations block cervix cancer growth through multiple mechanisms
Source: PLoS One. 2024 Sep 26;19(9):e0308233. doi: 10.1371/journal.pone.0308233 (PMC11426494; doi:10.1371/journal.pone.0308233)
Supplement: S3 Fig — Data shown represent the mean ± S.D. of at least three different preparations. *p < 0.05 vs. control (non-treated cells); **p < 0.05 vs. CXB or DMC. (DOCX) [file pone.0308233.s003.docx]

**
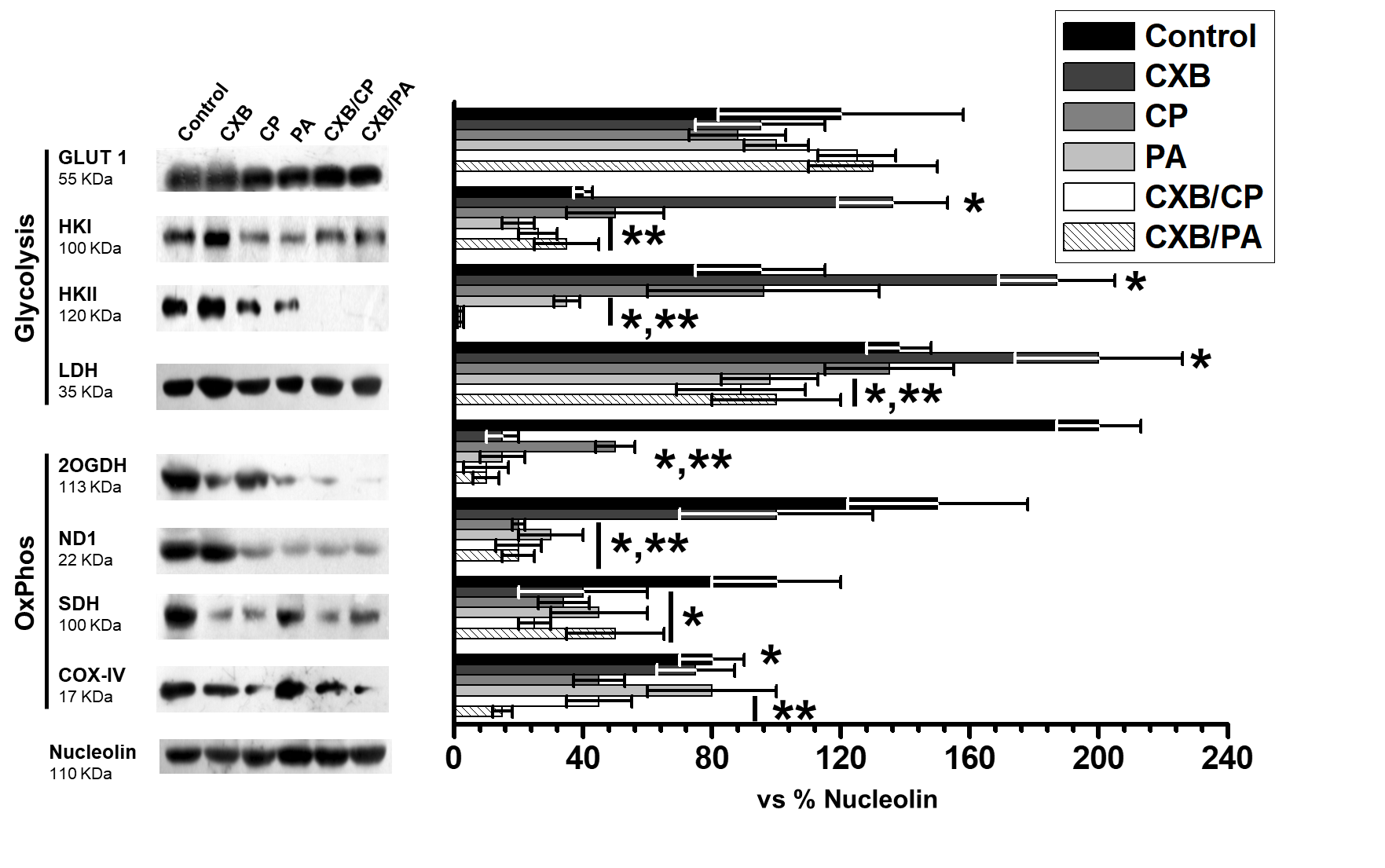
**

**S3 Fig. Effect of CXB and DMC combinations on the contents of glycolysis and OxPhos proteins in HeLa cells.** Data shown represent the mean ± S.D. of at least three different preparations. *p < 0.05 *vs*. control (non-treated cells); **p < 0.05 *vs*. CXB or DMC.
