# Supplementary material for: Synergistic celecoxib and dimethyl-celecoxib combinations block cervix cancer growth through multiple mechanisms
Source: PLoS One. 2024 Sep 26;19(9):e0308233. doi: 10.1371/journal.pone.0308233 (PMC11426494; doi:10.1371/journal.pone.0308233)
Supplement: S4 Fig — Data shown represent the mean ± S.D. of at least three different preparations. *p < 0.05 vs. control (non-treated cells). AUF, arbitrary units of fluorescence. (DOCX) [file pone.0308233.s004.docx]

**
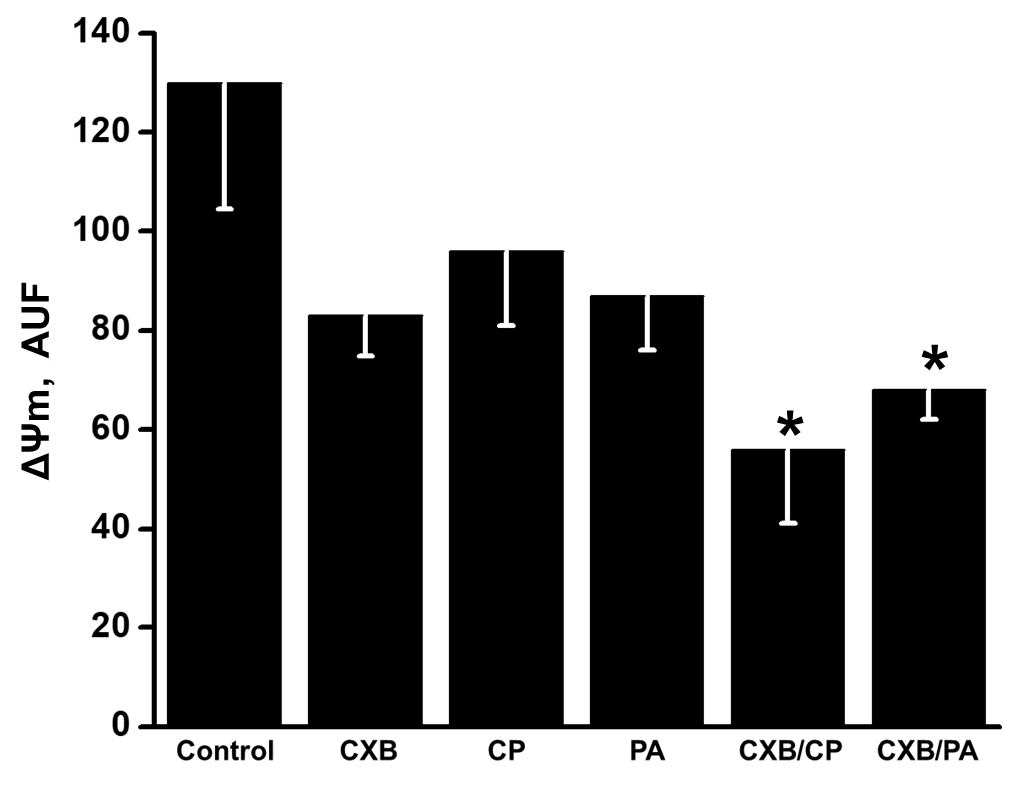
**

**S4 Fig. Effect of CXB combinations on the mitochondrial membrane potential (Δψm) in HeLa cells.** Data shown represent the mean ± S.D. of at least three different preparations. *p < 0.05 *vs*. control (non-treated cells). AUF, arbitrary units of fluorescence.
