# Supplementary material for: Synergistic celecoxib and dimethyl-celecoxib combinations block cervix cancer growth through multiple mechanisms
Source: PLoS One. 2024 Sep 26;19(9):e0308233. doi: 10.1371/journal.pone.0308233 (PMC11426494; doi:10.1371/journal.pone.0308233)
Supplement: S5 Fig — Epifluorescence images of HeLa (A, B) and SiHa cells (C) loaded with Mitotracker-green (MTG, 500 nM) and Lysotracker-red (LTR, 500 nM) in a complete DMEM medium after (A) CXB (n = 25), (B) DMC (n = 10) or (C) CXB/DMC (n = 10) treatment. Images were taken with the EVOS FL (Thermo Fisher Scientifc Waltham, MA, USA) cell imaging microscope using a 60×objective. Bars = 50 μm. White arrows indicate the mitochondria/lysosome dye co-loading. (DOCX) [file pone.0308233.s005.docx]

**A HeLa**

**
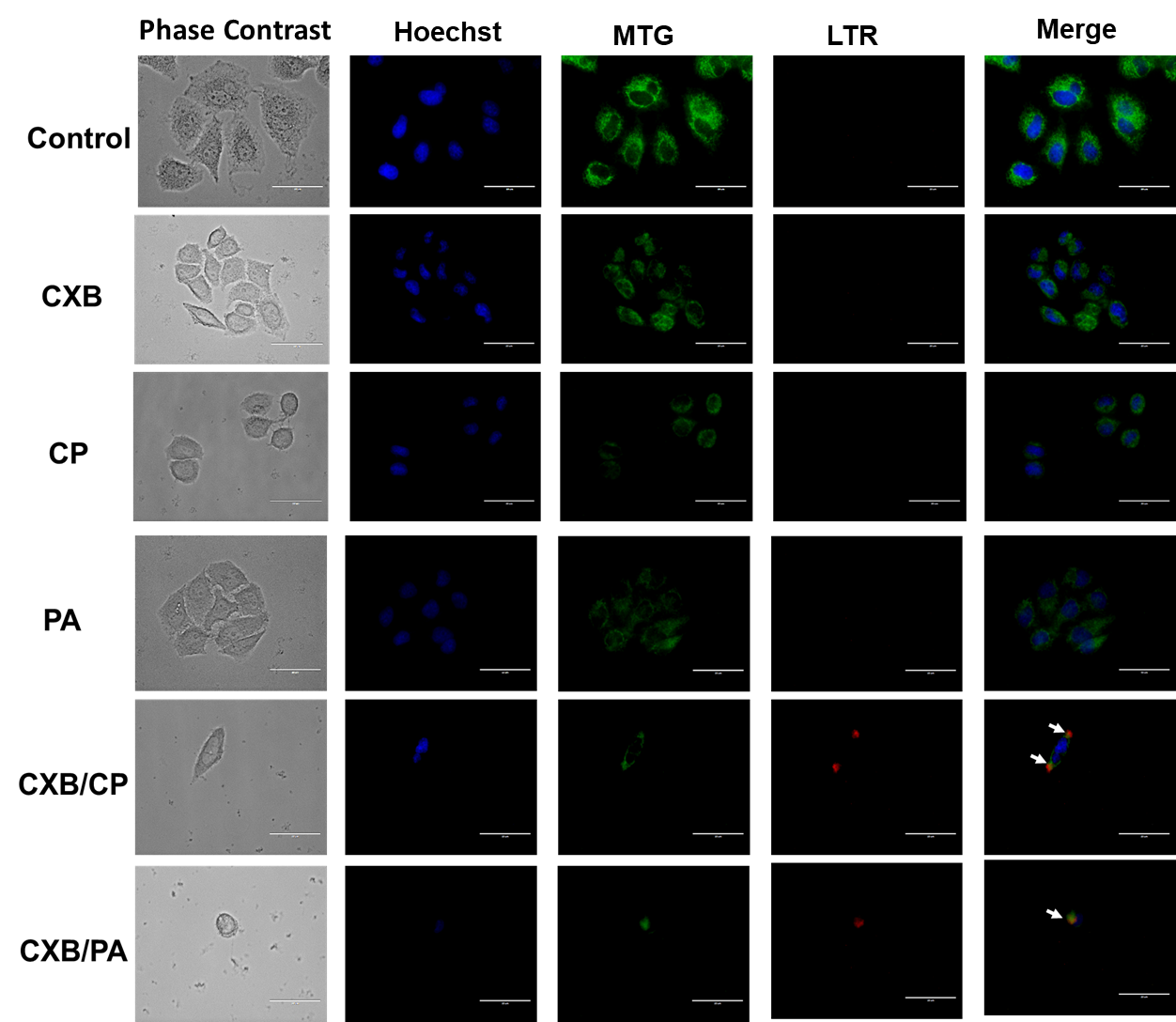
**

**B
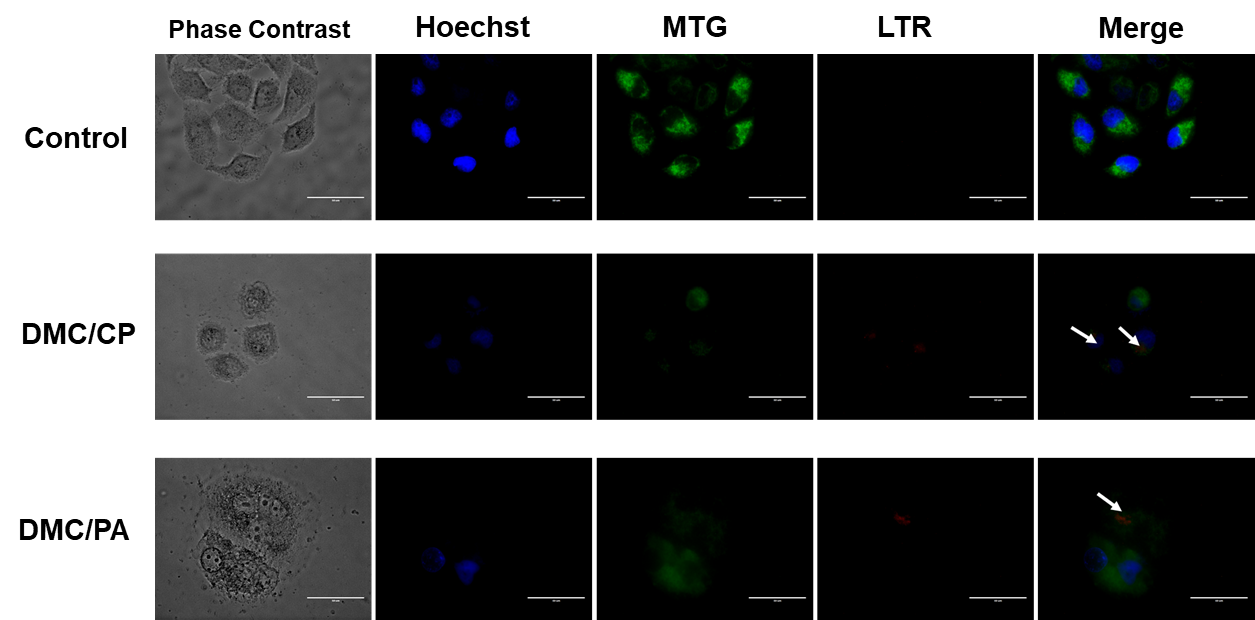
**

**
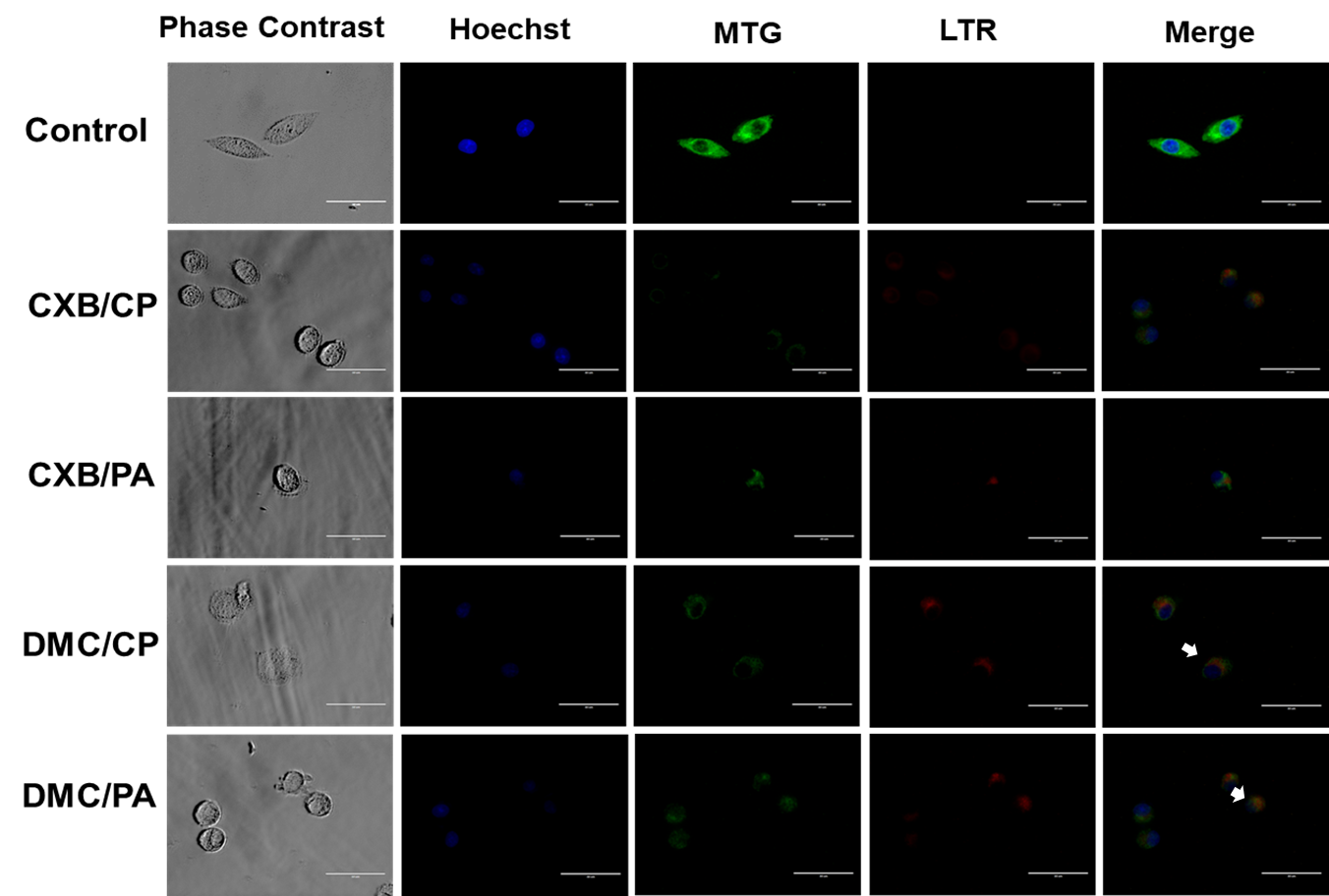
C SiHa**

**S52 Fig. Epifluorescence images of HeLa (A, B)** **and SiHa cells** **(C)** loaded with Mitotracker-green (MTG, 500 nM) and Lysotracker-red (LTR, 500 nM) in a complete DMEM medium after (A) CXB (n=25), (B) DMC (n=10) or (C) CXB/DMC (n=10) treatment. Images were taken with the EVOS FL (Thermo Fisher Scientifc Waltham, MA, USA) cell imaging microscope using a 60×objective. Bars=50 μm. White arrows indicate the mitochondria/lysosome dye co-loading.
