# Supplementary material for: Synergistic celecoxib and dimethyl-celecoxib combinations block cervix cancer growth through multiple mechanisms
Source: PLoS One. 2024 Sep 26;19(9):e0308233. doi: 10.1371/journal.pone.0308233 (PMC11426494; doi:10.1371/journal.pone.0308233)
Supplement: S6 Fig — Data shown represent the mean ± S.D. of at least three different preparations. *p < 0.05 vs. control (non-treated cells); **p < 0.05 vs. CXB. (DOCX) [file pone.0308233.s006.docx]

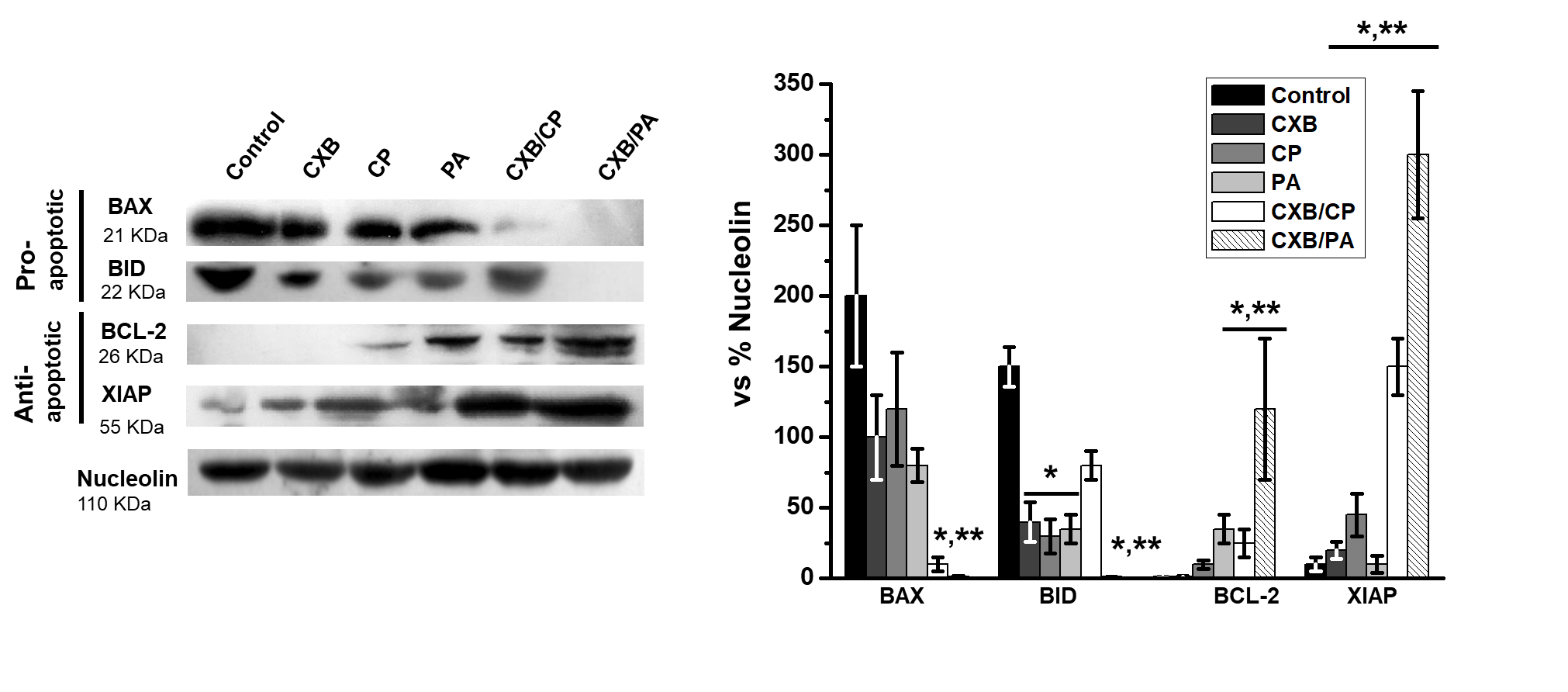


**S6 Fig. Effect of CXB combinations on pro- and anti-apoptotic protein contents in HeLa cells.** Data shown represent the mean ± S.D. of at least three different preparations. *p < 0.05 *vs*. control (non-treated cells); **p < 0.05 *vs*. CXB.
